# LAG3 immune inhibitors: a novel strategy for melanoma treatment

**DOI:** 10.3389/fonc.2024.1514578

**Published:** 2024-12-18

**Authors:** Renzheng Wu, Mingtang Zeng, Yuchen Zhang, Jianping He

**Affiliations:** ^1^ Department of Pharmacy, West China Hospital, Sichuan University, Chengdu, China; ^2^ Division of Abdominal Tumor Multimodality Treatment, Department of Medical Oncology, Cancer Center, West China Hospital, Sichuan University, Chengdu, China

**Keywords:** lymphocyte activation gene 3, immune checkpoint inhibitors, melanoma, antitumor immunity, clinical trial

## Abstract

Melanoma, a highly aggressive skin cancer, poses significant challenges in treatment, particularly for advanced or metastatic cases. While immunotherapy, especially immune checkpoint inhibitors (ICIs) targeting CTLA-4 and PD-1, has transformed melanoma management, many patients experience limited responses or develop resistance, highlighting the need for new therapeutic strategies. Lymphocyte activation gene 3 (LAG-3) has emerged as a promising target in cancer immunotherapy. LAG-3 inhibitors have shown potential in restoring T cell functions and enhancing anti-tumor immunity, particularly when used in combination with existing ICIs. This review discusses the latest advancements in LAG-3 inhibition for advanced melanoma, emphasizing its role in overcoming resistance and improving patient outcomes.

## 1 Introduction

Melanoma is one of the most aggressive forms of skin cancer, accounting for a significant proportion of skin cancer-related deaths worldwide (1). Despite advances in early detection and traditional therapies such as surgery, radiotherapy, and chemotherapy, the prognosis for patients with advanced or metastatic melanoma remains poor (2). These conventional approaches often fail to prevent recurrence and metastasis, highlighting the need for more effective treatments.

Over the past decade, immunotherapy has revolutionized melanoma treatment, particularly using immune checkpoint inhibitors (ICIs), which target immune regulatory pathways to enhance the body’s ability to combat cancer cells (3). Immune checkpoint inhibitors function by blocking the inhibitory signals that prevent T-cell activation, thereby allowing the immune system to attack tumor cells more effectively. CTLA-4 (cytotoxic T-lymphocyte-associated antigen 4) and PD-1 (programmed cell death protein 1) are two key immune checkpoint molecules targeted by ICIs. CTLA-4 modulates early T-cell activation in the lymph nodes by competing with CD28 for binding to the co-stimulatory molecules CD80 and CD86, leading to T-cell inhibition (4). In contrast, PD-1 functions later in the immune response, inhibiting T-cell activity in peripheral tissues by interacting with its ligands, PD-L1 and PD-L2, which are often expressed on tumor cells (5). The most well-established ICIs target CTLA-4 and PD-1, showing remarkable efficacy in many cases (6). However, a substantial portion of melanoma patients either do not respond to these therapies or develop resistance over time, reducing their long-term effectiveness (7). Furthermore, treatment-emergent adverse events (TRAEs) can pose significant challenges, limiting the use of ICIs in certain patient populations (8). These limitations underscore the importance of identifying and developing novel therapeutic targets to improve outcomes for melanoma patients.

Lymphocyte activation gene 3 (LAG-3) has recently emerged as a promising new target in cancer immunotherapy (9, 10). LAG-3 is an inhibitory receptor expressed on activated T cells and other immune cells, which plays a critical role in downregulating immune responses (11). Preclinical and clinical studies have shown that LAG-3 inhibitors can restore T cell function and enhance anti-tumor immunity, particularly in combination with other ICIs (12, 13). The synergy between LAG-3 and ICIs has become a key area of interest. While CTLA-4 and PD-1 inhibitors release the brakes on immune activation, LAG-3 inhibitors can further amplify the immune response by targeting additional inhibitory pathways. LAG-3 functions as a negative regulator, and its blockade can enhance the activity of both CTLA-4 and PD-1 inhibitors, potentially overcoming resistance mechanisms that limit the efficacy of these therapies (14). This combinatory approach has shown promise in preclinical models and early-phase clinical trials, suggesting that LAG-3 inhibitors may provide a complementary mechanism to enhance the effectiveness of existing ICIs in treating melanoma and other cancers.

As a result, LAG-3 inhibitors are now being explored in clinical trials for the treatment of advanced melanoma and other cancers. This review will examine the latest developments in LAG-3 inhibition as a therapeutic strategy for advanced melanoma, exploring its potential to overcome resistance to existing ICIs and improve patient outcomes.

## 2 LAG-3 structure and function

LAG-3 (Lymphocyte Activation Gene-3, CD223) is a type I transmembrane protein that functions as an immune inhibitory receptor by binding to MHC-II molecules, thereby modulating the proliferation and function of immune cells (15). The gene encoding LAG-3 is located on the distal short arm of chromosome 12, adjacent to the CD4 gene, with which it shares approximately 20% amino acid sequence similarity. LAG-3 consists of 498 amino acids and features an extracellular region spanning four immunoglobulin (Ig)-like domains (D1-D4), which plays a key role in its interaction with MHC-II molecules. Domain D1, the first immunoglobulin-like domain, contains an additional 30-amino acid loop, which enhances the binding affinity for MHC-II molecules on antigen-presenting cells (APCs), promoting effective suppression of immune responses (16). This structural feature has been considered crucial for LAG-3’s inhibitory function, as it helps to stabilize the interaction between LAG-3 and MHC-II. However, recent studies have introduced a new perspective. Ming et al. suggested that while the D1 loop enhances the affinity for MHC-II, its absence does not significantly alter the overall binding of LAG-3 to MHC-II, indicating that the 30-amino acid loop may not be as critical for overall binding affinity as previously thought (17). Domain D2 and D3 are believed to assist in stabilizing the extracellular portion of the receptor, ensuring that it is correctly oriented for efficient MHC-II binding. Recent studies have shown that antibodies directed against the D2 and D3 domains can block LAG-3 dimer formation and inhibit the binding of both MHC-II and FGL-1 ligands, suggesting a potential allosteric model of LAG-3 function that is tightly regulated by dimerization (18). The structural integrity of the receptor is further supported by Domain D4, which not only helps maintain the overall conformation of LAG-3 but also facilitates its interaction with other immune modulators, including co-receptors and ligands. However, an antibody targeting the D1 loop has been shown to block both MHC-II and FGL-1 binding and inhibit LAG-3’s suppressive function more potently than an antibody specific to Domain D4 (19).

LAG-3 can be cleaved by metalloproteinases between the D4 domain and the transmembrane region, generating soluble LAG-3 (sLAG-3), which may modulate its inhibitory functions by reducing LAG-3-mediated suppression. The intracellular tail of LAG-3 is responsible for signal transduction and contains three conserved motifs: a phosphorylatable serine (Ser484), the KIEELE motif, and a glutamate-proline (EP)-rich repeat sequence. Although the specific functions of these motifs are not fully understood, recent studies have shown that the EP motif plays a crucial role in TCR signal inhibition mediated by LAG-3, independent of MHC-II ligands (20, 21). At the immunological synapse, LAG-3 interacts with the TCR-CD3 complex, and the EP motif interferes with the binding of the Src family kinase Lck to CD4 and CD8 co-receptors by chelating zinc ions (Zn²^+^), thus blocking T cell activation signals (22). LAG-3 preferentially binds stable peptide-MHC-II complexes, although both LAG-3 and CD4 share overlapping binding sites on MHC-II, their distinct binding affinities create a competitive dynamic. This competition is less apparent *in vitro* due to these affinity differences, giving the appearance of non-competition in controlled experimental settings (23).

In addition to MHC-II, LAG-3 also binds to other ligands, such as liver sinusoidal endothelial cell lectin (LSECtin), Galectin-3 (Gal-3), and fibrinogen-like protein 1 (FGL1), which further inhibit T cell function and promote tumor immune evasion (24, 25). In an *in vitro* study of B16 melanoma cells, the binding of LAG-3 to LSECtin suppressed IFN-γ production and weakened the specific immune response against tumors, an effect that could be reversed by LAG-3 blockade (26). LAG-3 expression is induced on CD4 and CD8 T cells after antigen stimulation but is absent on naïve T cells. Prolonged antigen exposure leads to high levels of LAG-3 expression, resulting in the progressive impairment of T cell function, which is closely associated with regulatory T cell (Treg) activity (27). LAG-3 expression is significantly higher on inducible CD4CD25 Tregs than on effector memory T cells, and *in vitro* experiments have shown that anti-LAG-3 monoclonal antibodies can completely block the inhibitory effect of LAG-3-positive Tregs on CD4 T cell proliferation (28). *In vivo* studies further demonstrate that LAG-3 blockade weakens the protective role of Tregs in lethal pneumonia, indicating that LAG-3 plays a crucial role in mediating maximal inhibitory effects (28).

## 3 LAG-3 signaling pathway

**Figure 1:**
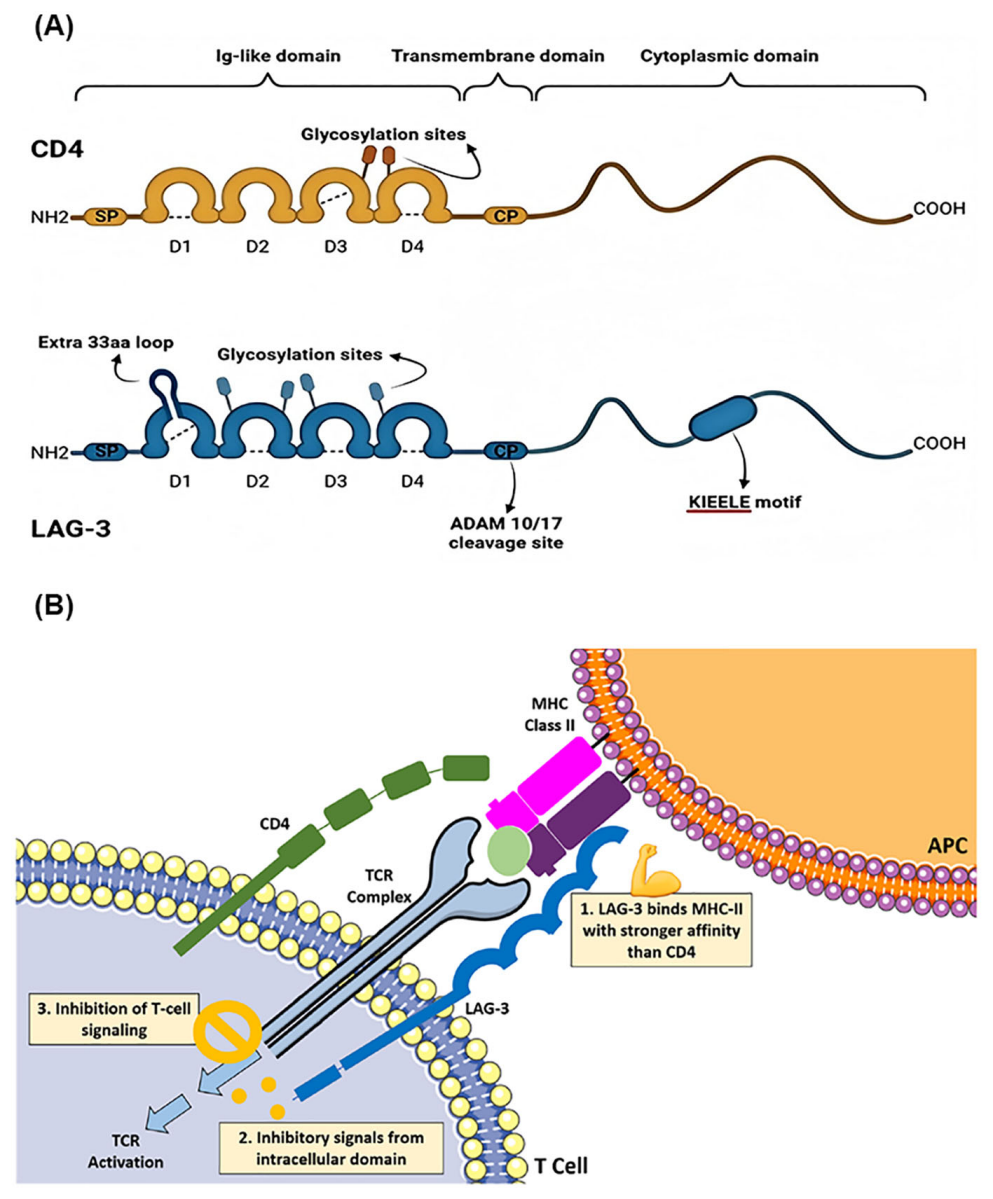
**(A)** The structural comparison and similarities between CD4 and LAG-3. Copyright 2023 Ibrahim et al. Biomedicines by PubMed Central. **(B)** Schematic overview of the LAG-3 signaling pathway. Copyright 2023 Kreidieh et al. Ther Adv Med Oncol by PubMed Central.

**Figure 1** depicts a schematic representation of the LAG-3 signaling pathway. LAG-3 is expressed on the cell membranes of tumor-infiltrating lymphocytes (TILs), activated CD4+ and CD8+ T cells, and regulatory T cells (Tregs), where it binds to MHC-II molecules on antigen-presenting cells (29). Although the interaction of LAG-3 with MHC-II is similar to that of CD4, it demonstrates a significantly higher affinity – approximately 100 times greater (30, 31). This interaction suppresses the binding of MHC-II to both CD4 and TCR, thereby inhibiting TCR signaling. The blockade of T cell activation by LAG-3 is not only due to competition with CD4, but also involves inhibitory signals transmitted through its intracellular domain (32). In addition, cross-linking of LAG-3 with CD3 attenuates T cell proliferation and cytokine secretion by impeding calcium influx. While the precise signaling mechanism of LAG-3 remains incompletely understood, its unique cytoplasmic tail, which differs from that of other immune checkpoints, suggests distinctive molecular properties and functional roles (28, 33). Further studies are needed to elucidate the full signaling pathway and potential therapeutic applications of targeting LAG-3 in a variety of malignancies and autoimmune diseases.

## 4 LAG-3 in melanoma immunotherapy

LAG-3 has emerged as a pivotal mediator of immunosuppression in melanoma, interacting with MHC-II molecules within the tumor microenvironment (TME) to induce tolerogenic plasmacytoid dendritic cells (pDCs) independently of Toll-like receptors (TLRs), thus sustaining an immunosuppressive milieu (34). LAG-3 and its ligands, Galectin-3 and MHC-II, exhibit high expression levels in melanoma lesions, particularly within inflamed T-cell phenotypes such as high-risk uveal melanoma, where the interaction between Galectin-3 and LAG-3 attenuates T-cell-mediated responses (35). Elevated pre-treatment serum levels of soluble LAG-3 are associated with resistance to anti-PD-1 therapy, correlating significantly with poorer disease control rate (DCR: p = 0.009) and reduced progression-free survival (PFS: p = 0.018). Furthermore, increased infiltration of LAG-3+ tumor-infiltrating lymphocytes in metastatic sites is linked to shorter PFS under PD-1 blockade (p = 0.07) (36). Hemon et al. further elucidated the functional and mechanistic roles of LAG-3, demonstrating that both LAG-3 and sLAG-3 transfected cells protect MHC-II-positive melanoma cells from FAS-induced and drug-induced apoptosis, primarily through activation of the MAPK/ERK and PI3K/AKT signaling pathways (37).

The co-targeting of LAG-3 with other immune checkpoint inhibitors has shown substantial promise in augmenting anti-tumor immunity. In models where CIITA, the master regulator of MHC-II, is expressed, dual anti-LAG-3/PD-1 ICI treatment has exhibited profound antitumor effects and inhibited tumor growth in mice, resulting in stronger IFN-γ production and enhanced cytotoxicity of CD8+ T cells (38). In the B16 melanoma model, the dual depletion of LAG-3 and PD-1 on TILs reduced tumor-induced tolerance and achieved higher response rates compared to single-checkpoint deficient mice, with an 80% tumor elimination rate compared to 40% in PDCD1-deficient mice and no control in wild-type and LAG-3-deficient mice. Peripheral blood profiling of melanoma patients eligible for immunotherapy revealed that those with the LAG-3 immunotype had poorer outcomes following ICI treatment compared to patients with the LAG-3^+−^ immunotype (median overall survival: 22.2 vs. 75.8 months, respectively; p = 0.031), irrespective of other immune biomarkers such as PD-1 and tumor mutational burden (TMB) (39). Relatlimab, a LAG-3 inhibitor, has been employed in the treatment of advanced melanoma, with ongoing clinical trials evaluating various LAG-3 inhibitors, underscoring the potential of LAG-3 as a promising therapeutic target in melanoma treatment.

## 5 LAG-3 immune checkpoint inhibitors for melanoma

Within the tumor microenvironment, LAG-3 expression is markedly upregulated, particularly in advanced melanoma, where it acts on tumor-infiltrating CD4CD25 high FoxP3 Treg subsets (40). These cells selectively suppress immune responses in tumor-invaded lymph nodes and metastatic lesions by secreting immunosuppressive cytokines. T cells lacking LAG-3 can bypass Treg suppression and differentiate into Th1 cells, thereby enhancing anti-tumor responses. To counteract LAG-3-mediated immune suppression, researchers have developed various LAG-3 inhibitors that block the interaction between LAG-3 and its ligands, restoring T cell function and showing remarkable efficacy in cancer treatment (23, 25). The main forms of LAG-3 inhibitors include LAG-3 monoclonal antibodies, soluble LAG-3 fusion proteins, and bispecific antibodies (41). Most anti-LAG-3 monoclonal antibodies are humanized IgG4 antibodies, except for Etigilimab, which is an IgG1 antibody. Eftilagimod alpha (IMP321) is the only identified soluble LAG-3 fusion protein. Bispecific antibodies are also under exploration, showing great therapeutic potential (42). The development of these inhibitors offers new therapeutic strategies in the fight against tumor immune evasion.

Comparative analyses between LAG-3 inhibitors and other emerging therapies provide valuable insights into their positioning within the current melanoma treatment landscape. PD-1 inhibitors such as pembrolizumab and nivolumab have set a high benchmark by significantly improving survival rates in melanoma patients. Studies have shown a median overall survival of 32.7 months for nivolumab compared to 19.9 months for ipilimumab, a CTLA-4 inhibitor (43, 44). However, not all patients respond to PD-1 inhibitors, and resistance can develop, highlighting the need for alternative or complementary approaches. LAG-3 inhibitors, such as Relatlimab, have emerged as promising candidates in this context. In a pivotal clinical trial, the combination of Relatlimab and nivolumab demonstrated a significant improvement in progression-free survival (PFS) compared to nivolumab alone, with a median PFS of 10.1 months versus 4.6 months, respectively (45). This combination leverages the distinct mechanisms of LAG-3 and PD-1 pathways, offering a synergistic effect that can potentially overcome the limitations observed with monotherapy. Additionally, other emerging therapies, such as tumor-infiltrating lymphocyte (TIL) therapy and novel bispecific antibodies, are also showing promise. TIL therapy, for instance, has demonstrated durable responses in patients with advanced melanoma, with response rates around 36% and some patients achieving long-term remission (46). On the other hand, bispecific antibodies that simultaneously target multiple immune checkpoints are being investigated for their ability to enhance immune responses more effectively than single-agent therapies (47). By integrating LAG-3 inhibitors with these emerging therapies, researchers hope to address the multifaceted nature of tumor immune evasion. For example, combining LAG-3 inhibitors with TIL therapy or bispecific antibodies could potentially enhance the anti-tumor efficacy by engaging different aspects of the immune system. This strategic combination could provide a more comprehensive attack on the tumor, leading to improved outcomes for patients who have limited response to current treatments.

In conclusion, while PD-1 inhibitors remain a cornerstone in melanoma therapy, the addition of LAG-3 inhibitors, either alone or in combination with other emerging treatments, represents a significant advancement. These combinations not only have the potential to enhance efficacy and overcome resistance but also to broaden the therapeutic options available to melanoma patients, ultimately improving overall prognosis and survival rates.

### 5.1 Monoclonal antibody

#### 5.1.1 Relatlimab

Relatlimab, a humanized IgG4 monoclonal antibody developed by Bristol-Myers Squibb (BMS), targets LAG-3, inhibiting its interaction with MHC-II and thereby restoring T cell function to attack and eliminate tumor cells. As the first FDA-approved LAG-3 inhibitor for metastatic melanoma treatment (48), Relatlimab represents a significant advancement in immunotherapy. The Phase III RELATIVITY-047 trial was conducted to assess the efficacy of combining Relatlimab with Nivolumab versus Nivolumab monotherapy in 714 treatment-naïve patients with metastatic melanoma. Participants were randomized to receive either 480 mg of Nivolumab every four weeks or a combination of 480 mg Nivolumab and 160 mg Relatlimab via intravenous injection. With a median follow-up of 13.2 months, the trial reported that the median progression-free survival (PFS) for the combination therapy group was 10.1 months, significantly surpassing the 4.6 months observed in the Nivolumab monotherapy group. This PFS advantage was consistent across all major subgroups (45). **Table 1** provides an overview of the clinical trials evaluating the efficacy of Relatlimab in targeting LAG-3 for the treatment of melanoma. Moreover, the safety profile of the Relatlimab and Nivolumab combination therapy was favorable. The incidence of Grade 3/4 treatment-related adverse events was 18.9%, considerably lower than the 55% observed with the Nivolumab and Ipilimumab combination therapy for melanoma (49). Ongoing studies are investigating the efficacy of Relatlimab in resectable melanoma and other clinical settings. In a neoadjuvant clinical trial for resectable melanoma (NCT02519322), the Relatlimab and Nivolumab regimen achieved a complete remission (CR) rate of 57% among 30 patients in the intent-to-treat (ITT) population, with an overall pathological response rate of 70%, and no reported Grade 3/4 TRAEs. Among patients exhibiting any pathological response, the one-year and two-year recurrence-free survival (RFS) rates were 100% and 92%, respectively, while those without a pathological response had RFS rates of 88% and 55% (50). These results suggest that the combination of Relatlimab and Nivolumab offers improved efficacy and safety over previous treatment options for patients with advanced melanoma.

**Table 1:** Relatlimab targets LAG-3 in clinical trials for melanoma.

ClinicalTrials.gov Identifier	Phase	Indication	Intervention	Target
NCT01968109	I/IIa	Immunotherapy-naïve and immunotherapy-experienced advanced solid tumors	Single-agent Relatlimab or Relatlimab + Nivolumab	Anti-LAG-3 or Anti-LAG-3 + Anti-PD-1
NCT02465060	II	Solid tumors, including melanoma, that have progressed after at least one line of standard treatment or for which no established treatment approach exists	Subprotocol Z1M (LAG-3 expression ≥ 1%); Relatlimab + Nivolumab	Anti-LAG-3 + Anti-PD-1
NCT03470922	II/III	Previously untreated metastatic or unresectable melanoma	Relatlimab + Nivolumab vs. Nivolumab	Anti-LAG-3 + Anti-PD-1 vs. Anti-PD-1
NCT03743766	II	Metastatic melanoma naïve to prior immunotherapy in a metastatic setting	Nivolumab, Relatlimab, or Nivolumab + Relatlimab followed by Nivolumab + Relatlimab	Anti-PD-1, Anti-LAG-3 or Anti-LAG-3 + Anti-PD-1
NCT03978611	I/IIa	Unresectable or metastatic melanoma, after progression on anti-PD-1 ICI therapy	Relatlimab + Ipilimumab	Anti-LAG-3 + Anti-CTLA-4
NCT04552223	II	Metastatic uveal melanoma	Relatlimab + Nivolumab	Anti-LAG-3 + Anti-PD-1
NCT04935229	I	Metastatic uveal melanoma in the liver	Pressure-enabled hepatic artery infusion of SD-101, alone or in combination with Nivolumab, Nivolumab + Ipilimumab, or Relatlimab + Nivolumab	PEDD/HAI TLR9-agonist +/- Anti-PD-1, Anti-PD-1 + Anti-CTLA-4 or Anti-LAG-3 + Anti-PD-1
NCT05002569	III	Adjuvant therapy for completely resected clinical-stage-III–IV melanoma	Relatlimab + Nivolumab vs. Nivolumab	Anti-LAG-3 + Anti-PD-1 vs. Anti-PD-1
NCT05077280	II	Metastatic uveal melanoma	Concurrent stereotactic radiotherapy with Relatlimab + Nivolumab	SBRT with Anti-LAG-3 + Anti-PD-1
NCT05418972	II	Neoadjuvant +/− adjuvant therapy for high risk, clinical-stage-II cutaneous melanoma	pre-surgery +/− post-surgery Relatlimab + Nivolumab	Anti-LAG-3 + Anti-PD-1
NCT05428007	II	Unresectable clinical-stage-III–IV melanoma	Sarilumab, Ipilimumab and Relatlimab + Nivolumab	Anti-IL-6R, Anti-CTLA-4 and Anti-LAG-3 + Anti-PD-1
NCT05625399	III	Previously untreated metastatic or unresectable melanoma	Relatlimab + Nivolumab	Anti-LAG-3 + Anti-PD-1
NCT05629546	I	Advanced or metastatic melanoma, after progression on ICI therapy	Memory-like natural killer cells (autologous or allogeneic) with Relatlimab + Nivolumab	ML NKs with Anti-LAG-3 + Anti-PD-1
NCT05704647	II	Active melanoma brain metastases	Relatlimab + Nivolumab	Anti-LAG-3 + Anti-PD-1
NCT05704933	I	Neoadjuvant therapy for surgically resectable melanoma brain metastases	Relatlimab + Nivolumab vs. Nivolumab + Ipilimumab	Anti-LAG-3 + Anti-PD-1 vs. Anti-PD-1 + Anti-CTLA-4

#### 5.1.2 Fianlimab

Fianlimab, a humanized anti-LAG3 monoclonal antibody labeled with Zr-89, was initially developed by Regeneron for PET imaging of tumors. However, subsequent investigations revealed its potential to inhibit the LAG3/MHC-II interaction, thereby enhancing T cell-mediated cytotoxicity and suppressing tumor cell proliferation (51). In a Phase I clinical trial (NCT03005782) evaluating the combination of Fianlimab with the anti-PD-1 antibody Cemiplimab in patients with advanced melanoma, 98 participants were administered Fianlimab (1600 mg) and Cemiplimab (350 mg) every three weeks over a period of 12 months. The trial outcomes indicated an overall objective response rate (ORR) of 61.2%, with 12 complete responses (CR) and 48 partial responses (PR), and a median progression-free survival (PFS) of 15.3 months (52). Moreover, a Phase III clinical trial (NCT05352672) was launched on April 6, 2022, to assess the efficacy of the Fianlimab and Cemiplimab combination compared to Pembrolizumab in treatment-naïve patients with advanced melanoma. The results of this ongoing study are anticipated to provide further insights into the therapeutic potential of Fianlimab in melanoma treatment.

#### 5.1.3 LBL-007

LBL-007 is a humanized IgG4 monoclonal antibody developed by Nanjing Weilizhibo Company, specifically targeting LAG3. Its primary mechanism of action involves blocking the interaction between LAG3 and MHC-II molecules, thereby restoring T cell-mediated cytotoxicity against tumor cells (53). In a Phase I clinical trial (NCT04640545), researchers evaluated the efficacy of LBL-007 in combination with Toripalimab for patients with advanced or metastatic melanoma, irrespective of their prior exposure to anti-PD-1 therapies. The trial enrolled 37 patients, 32 of whom were assessed for efficacy. The overall response rate (ORR) across the cohort was 12.5%, while the disease control rate (DCR) was 53.1%. Notably, the ORR was 27.3% for acral melanoma and 0% for mucosal melanoma in patients who had not previously received anti-PD-1 therapy. The corresponding DCRs were 81.8% for acral melanoma and 50.0% for mucosal melanoma. In contrast, patients resistant to prior anti-PD-1 therapy showed a DCR of 18.2 percent. Progression-free survival (PFS) and overall survival (OS) metrics are still under observation (54). These preliminary findings suggest that the combination of LBL-007 and Toripalimab holds therapeutic potential, especially for melanoma patients who have developed resistance to PD-1 inhibitors. The differentiation in response rates between the acral and mucosal melanoma subtypes underscores the need for further stratified analyses to optimize treatment strategies. Future studies with larger cohorts and longer follow-up periods will be crucial to validate these results and to elucidate the long-term benefits and safety profile of this combination therapy in melanoma treatment.

#### 5.1.4 INCAGN2385

Incagn-2385 is an innovative monoclonal antibody developed through a strategic collaboration between Incyte and Agenus, specifically designed to target LAG-3, a crucial immune checkpoint that plays a significant role in regulating immune responses (55). By inhibiting LAG-3, Incagn-2385 aims to enhance the body’s immune system in recognizing and attacking cancer cells, potentially leading to improved therapeutic outcomes for patients with advanced malignancies. The initial findings from a phase I clinical trial (NCT03538028) have been encouraging, demonstrating not just the safety and tolerability of Incagn-2385, but also suggesting promising efficacy in the treatment of various advanced cancers. These positive results have paved the way for further exploration into the full potential of this novel therapeutic agent. Currently, a phase Ib/II clinical trial (NCT04370704) is underway, focusing on the synergistic effects of Incagn-2385 when combined with other immune-modulating therapies. This trial is particularly noteworthy as it investigates the combination of Incagn-2385 with the TIM-3 antibody INCAGN02390 and the anti-PD-1 antibody INCMGA00012. The objective of this combination therapy is to leverage multiple mechanisms of action to enhance anti-tumor immunity, particularly in patients with advanced melanoma, a notoriously challenging cancer to treat. As the trial progresses, it holds the promise of not only improving patient outcomes but also contributing valuable insights into the evolving landscape of cancer immunotherapy.

#### 5.1.5 Ieramlimab (LAG525)

Ieramlimab (LAG525) is a humanized IgG4 monoclonal antibody developed by Novartis that inhibits the interaction between LAG3 and MHC-II, as well as FGL-1, thereby suppressing tumor cell growth. Notably, crystallographic analysis has revealed that the amino acid motifs in the BC and DE loops of the LAG3 binding site of Ieramlimab are located at a considerable distance from the hot spot “extra loop,” while the RGD motif, critical for MHC-II binding, appears spherical in structure (56). These findings suggest that Ieramlimab may possess a novel binding epitope, providing new insights into the mechanism of LAG3-mediated antitumor action. The combination of Ieramlimab and Spartalizumab has demonstrated significant antitumor activity in both solid tumors and hematological malignancies (57). Spartalizumab is a monoclonal antibody targeting programmed cell death protein 1 (PD-1), a checkpoint receptor found on T cells that plays a critical role in suppressing immune responses (58). PD-1 interacts with its ligands, PD-L1 and PD-L2, which are often upregulated on tumor cells and within the tumor microenvironment, leading to T cell exhaustion and impaired immune surveillance. By blocking the PD-1 pathway, Spartalizumab prevents this inhibitory interaction, thereby enhancing T-cell activation and restoring the immune system’s ability to recognize and attack tumor cells (59). This mechanism reduces immunosuppression within the TME and promotes a more effective anti-tumor immune response. In a Phase I clinical trial (NCT02460224) comparing 42 patients with advanced solid tumors receiving Ieramlimab in combination with Spartalizumab versus those treated with Spartalizumab alone, the combination group exhibited a complete response (CR) rate of 15% and a progression-free survival (PFS) of 2 years, with favorable safety profiles (60). Additionally, a Phase II clinical trial (NCT03484923) evaluating the efficacy of Ieramlimab combined with Spartalizumab in patients with PD-1 inhibitor-resistant melanoma indicated promising antitumor effects against LAG3-positive metastatic melanoma (61). These results suggest that Spartalizumab in combination with Ieramlimab has demonstrated good safety and tolerability in patients with resistant melanoma, positioning it as a potential new therapeutic option.

### 5.2 Bispecific antibody

#### 5.2.1 RO-7247669

RO-7247669 is an innovative bispecific antibody developed by Roche that uniquely targets both LAG-3 and PD-1, two critical immune checkpoints involved in the regulation of anti-tumor immunity (21). By simultaneously inhibiting these pathways, RO-7247669 aims to enhance the immune system’s ability to recognize and combat cancer cells, thereby exerting significant antitumor effects. In a phase I clinical trial (NCT04140500) designed to evaluate the efficacy of RO-7247669 in patients with advanced solid tumors, including challenging malignancies such as melanoma, esophageal squamous cell carcinoma, and non-small cell lung cancer, the results were promising (62). Among the 35 patients treated, the trial reported an overall response rate (ORR) of 17.1%, alongside a disease control rate (DCR) of 51.4%. These results suggest that RO-7247669 may offer a viable therapeutic option for patients with difficult-to-treat tumors, indicating its potential role in advancing cancer therapy. Building on these encouraging findings, a phase II clinical trial (NCT05419388) is currently in progress, actively recruiting participants with previously untreated, unresectable, or metastatic melanoma. This trial is designed to further assess the efficacy, safety, and pharmacokinetics of RO-7247669 in multiple doses with the goal of identifying an optimal dose for subsequent clinical development. As the study unfolds, it holds a significant promise for enhancing treatment strategies for melanoma and potentially other advanced cancers, marking a step forward in the quest for more effective immunotherapies.

#### 5.2.2 XmAb22841 (Bavunalimab)

XmAb22841 is a cutting-edge bispecific antibody developed by Xencor, designed to target both LAG-3 and CTLA-4 (Cytotoxic T-Lymphocyte Associated Protein 4), two critical immune checkpoints that regulate the immune system’s ability to mount an effective antitumor response (63). By simultaneously inhibiting these pathways, XmAb22841 aims to significantly enhance the body’s immune response against tumors, offering a promising strategy for cancer treatment. Two clinical trials are currently underway to evaluate the therapeutic potential of XmAb22841. One of these is a phase Ib/II trial (NCT05695898) that is specifically investigating the safety, tolerability, and pharmacokinetics of escalating doses of XmAb22841 in combination with the anti-PD-1 antibody XmAb23104. The trial focused on patients with metastatic melanoma, a particularly aggressive form of skin cancer that often proves resistant to conventional treatment. By exploring this combination therapy, researchers hope to better understand how these agents can work together to enhance anti-tumor efficacy and improve patient outcomes.

#### 5.2.3 EMB-02

EMB-02 is a novel bispecific antibody engineered to simultaneously target LAG-3 and PD-1, developed by EpimAb Biotherapeutics. Extensive preclinical studies have revealed its potent anti-tumor efficacy, particularly in tumor models that exhibit resistance to conventional anti-PD-1 therapies. This promising therapeutic candidate is now undergoing rigorous evaluation in a Phase I/II clinical trial (NCT04618393), which aims to assess its safety, tolerability, and preliminary efficacy in patients with advanced solid tumors, including locally advanced/metastatic melanoma with >1 prior therapy (PD-1/L1 +/− CTLA-4 ICI).

#### 5.2.4 Tebotelimab

Tebotelimab (MGD-013) is a human IgG4κ bispecific antibody targeting PD-1 and LAG3, developed by MacroGenics. It primarily functions by blocking the interactions between PD-1 and its ligands PD-L1 and PD-L2, as well as LAG3 and MHC-II, thereby restoring T cell functionality and enhancing anti-tumor responses. Clinical trials have demonstrated its promising safety and anti-tumor efficacy in melanoma, gastric cancer, and colon cancer (64–66). In a Phase I clinical trial (NCT04653038) evaluating the safety and efficacy of Tebotelimab in patients with malignant melanoma, 25 participants received intravenous injections of 600 mg of Tebotelimab every two weeks. The results indicated an overall response rate (ORR) of 24% and a disease control rate (DCR) of 40%. However, 44% of patients experienced immune-related adverse events (irAEs), with the most common being hypothyroidism, hyperthyroidism, and leukopenia. Additionally, 28% of patients experienced grade 3 or 4 treatment-related adverse events, with treatment interruptions and fatalities attributable to TRAEs occurring in 4% of cases (67). These findings suggest that Tebotelimab demonstrates significant anti-tumor activity in the treatment of melanoma, while highlighting the need for careful monitoring of immune-related side effects.

### 5.3 Fusion protein

Eftilagimod alpha (IMP321) is a soluble LAG3 fusion protein developed by Immutep, created by fusing the extracellular domain of LAG3 with the Fc region of human immunoglobulin. As a novel immune checkpoint inhibitor (ICI), its mechanism primarily involves binding to MHC-II molecules on antigen-presenting cells, leading to the activation of CD8+ T cells and enhancement of the immune response. This approach has potential therapeutic applications in cancer, hepatitis B, and influenza (31). In a Phase I clinical trial evaluating the efficacy of IMP321 in combination with MART-1 for the treatment of patients with stage IV melanoma who received PBMC transplantation, the study revealed that while the overall response rate (ORR) was only 17%, IMP321 significantly stimulated CD8+ T cells to secrete higher levels of IFN-γ, TNF-α, and IL-2, indicating a more durable anti-tumor immune response (68). Additionally, a Phase I clinical trial (NCT02676869) assessed the efficacy of IMP321 in combination with Pembrolizumab for metastatic melanoma, stratifying patients based on the subcutaneous dosage of IMP321 into Group A (6 mg) and Group B (30 mg). Eighteen patients were included in group A of the study. All patients had stage IV melanoma. Disease stage at baseline was M1c in 14 patients (77.8%), M1b in 3 patients (16.7%) and M1a in 1 patient (5.6%). Patients were required to have asymptomatic immune-related progressive disease (ie, slowly progressive disease, not requiring urgent intervention and stable performance status) or suboptimal response (ie, immune-related stable disease (irSD) or partial response (irPR)) after three cycles (≈9 weeks) of pembrolizumab. Group B was a six-patient toxicity lead-in cohort. All patients had stage IV melanoma. The stage of disease was M1c in four patients (66.7%) and M1b in two patients (33.3%). Most patients (83.3%) had undergone prior anticancer surgery; one (16.7%) had received prior anticancer radiation; none had received prior systemic anticancer medication. The results showed no dose-limiting toxicities (DLTs) associated with IMP321. In Group A, the ORR was 33% and the disease control rate (DCR) was 55.6%, and the ORR was 50% with a DCR of 83% in Group B. Furthermore, it was observed that IMP321 could promote a sustained increase in CD8+ and CD4+ T cells within the tumor microenvironment by activating dendritic cells (DCs), thereby enhancing tumor immune surveillance (69). These results suggest that the combination of IMP321 with other therapies demonstrates favorable safety and efficacy in the treatment of melanoma.

## 6 Summary and outlook

The development of LAG-3 inhibitors has garnered widespread attention globally, particularly after the FDA approval of Relatlimab for melanoma treatment. As a newly discovered inhibitory checkpoint, LAG-3 plays a crucial role in regulating T cell homeostasis by inhibiting T cell activation, proliferation, cytokine secretion, and effector function. The emergence of immune checkpoint therapies, especially those targeting PD-1 and CTLA-4, has revolutionized cancer treatment, and LAG-3 is expected to become an important therapeutic target in oncology. Despite the promising safety profile and anti-tumor activity of LAG-3-targeted molecules in multiple clinical studies, their efficacy as monotherapy remains limited, making combination therapy a necessary approach. However, combination therapies also significantly increase the risk of treatment-related adverse events. Moreover, the precise anti-tumor mechanisms of LAG-3 inhibitors are not fully elucidated, which partially hinders their further development. Therefore, future research should focus on several key areas.

First, immune checkpoints such as PD-1 and CTLA-4 interact to form a complex immune suppression network, making their anti-tumor mechanisms more difficult to predict and understand. Therefore, future studies should focus on deeply exploring the specific mechanisms of action of LAG-3 inhibitors, including their roles within the tumor microenvironment and their interactions with other immune checkpoints and immune cells. By understanding these mechanisms, researchers can develop more targeted treatment strategies to improve the efficacy of LAG-3 inhibitors (70). Second, the identification of biomarkers is another crucial direction for the future development of LAG-3 inhibitors. Although some clinical trials have demonstrated the anti-tumor potential of LAG-3 inhibitors, not all patients benefit from them. Developing biomarkers to predict which patients are most likely to respond positively to LAG-3 inhibitors could significantly improve treatment outcomes and reduce unnecessary side effects. Biomarkers can help select appropriate patient populations, thereby optimizing treatment regimens (71). For instance, genomics, transcriptomics, or proteomics approaches could be employed to identify molecular markers associated with the efficacy of LAG-3 inhibitors, which may become a major focus of future research. Another important research direction is the mechanism of resistance to LAG-3 inhibitors. Although LAG-3 inhibitors have shown some success in clinical practice, a subset of patients still develops resistance, potentially limiting their widespread application. Investigating how tumor cells develop resistance through various mechanisms and uncovering immune evasion strategies will provide new approaches for overcoming resistance and improving treatment outcomes. Combining LAG-3 inhibitors with other immunotherapies or targeted therapies could be an important path for overcoming resistance and enhancing efficacy (30).

In conclusion, although LAG-3 inhibitors hold tremendous potential in cancer immunotherapy, future research faces numerous challenges. Understanding their anti-tumor mechanisms, identifying biomarkers, and overcoming resistance will be the core issues in the field. As these challenges are addressed, LAG-3 inhibitors are expected to bring new breakthroughs in the treatment of melanoma and other malignancies, ultimately improving patient prognosis.
